# Binding of Host Cell Surface Protein Disulfide Isomerase by Anaplasma phagocytophilum Asp14 Enables Pathogen Infection

**DOI:** 10.1128/mBio.03141-19

**Published:** 2020-01-28

**Authors:** Ryan S. Green, Waheeda A. Naimi, Lee D. Oliver, Nathaniel O’Bier, Jaehyung Cho, Daniel H. Conrad, Rebecca K. Martin, Richard T. Marconi, Jason A. Carlyon

**Affiliations:** a Department of Microbiology and Immunology, School of Medicine, Virginia Commonwealth University Medical Center, Richmond, Virginia, USA; b Department of Pharmacology, College of Medicine, University of Illinois at Chicago, Chicago, Illinois, USA; Yale University School of Medicine

**Keywords:** adhesin, *Anaplasma phagocytophilum*, obligate intracellular bacteria, protein disulfide isomerase, host-pathogen interactions

## Abstract

Diverse intracellular pathogens rely on eukaryotic cell surface disulfide reductases to invade host cells. Pharmacologic inhibition of these enzymes is cytotoxic, making it impractical for treatment. Identifying and mechanistically dissecting microbial proteins that co-opt surface reductases could reveal novel targets for disrupting this common infection strategy. Anaplasma phagocytophilum invades neutrophils by an incompletely defined mechanism to cause the potentially fatal disease granulocytic anaplasmosis. The bacterium’s adhesin, Asp14, contributes to invasion by virtue of its C terminus engaging an unknown receptor. Yeast-two hybrid analysis identified protein disulfide isomerase (PDI) as an Asp14 binding partner. Coimmunoprecipitation confirmed the interaction and validated it to be Asp14 C terminus dependent. PDI knockdown and antibody-mediated inhibition of PDI reductase activity impaired A. phagocytophilum infection of but not binding to host cells. Infection during PDI inhibition was rescued when the bacterial but not host cell surface disulfide bonds were chemically reduced with tris(2-carboxyethyl)phosphine-HCl (TCEP). TCEP also restored bacterial infectivity in the presence of an Asp14 C terminus blocking antibody that otherwise inhibits infection. A. phagocytophilum failed to productively infect myeloid-specific-PDI conditional-knockout mice, marking the first demonstration of *in vivo* microbial dependency on PDI for infection. Mutational analyses identified the Asp14 C-terminal residues that are critical for binding PDI. Thus, Asp14 binds and brings PDI proximal to A. phagocytophilum surface disulfide bonds that it reduces, which enables cellular and *in vivo* infection.

## Abstract (precis)

Anaplasma phagocytophilum infects neutrophils to cause granulocytic anaplasmosis, an emerging potentially fatal disease and the second-most common tick-borne illness in the United States. Treatment options are limited, and no vaccine exists. Due to the bacterium’s obligatory intracellular lifestyle, A. phagocytophilum survival and pathogenesis are predicated on its ability to enter host cells. Understanding its invasion mechanism will yield new targets for preventing bacterial entry and, hence, disease. We report a novel entry pathway in which the A. phagocytophilum outer membrane protein Asp14 binds host cell surface protein disulfide isomerase via specific C-terminal residues to promote reduction of bacterial surface disulfide bonds, which is critical for cellular invasion and productive infection *in vivo*. Targeting the Asp14 C terminus could be used to prevent/treat granulocytic anaplasmosis. Our findings have broad implications, as a thematically similar approach could be applied to block infection by other intracellular microbes that exploit cell surface reductases.

## INTRODUCTION

Intracellular microbes are significant causes of morbidity and mortality. As host cell entry is essential for infection by any intracellular pathogen, dissecting host-microbe interactions that facilitate pathogen invasion is critically important. Mechanistic insights yielded by such studies can benefit the development of novel strategies for treating and preventing infection. Anaplasma phagocytophilum is an *Ixodes* species tick-transmitted obligate intracellular bacterium that infects neutrophils to cause the emerging zoonosis known as granulocytic anaplasmosis in humans and some domestic animals (1, 2). Human granulocytic anaplasmosis (HGA) can also be transmitted perinatally, via blood transfusion, and possibly, by exposure to infected blood (3–8). HGA manifestations include fever, chills, headache, malaise, leukopenia, thrombocytopenia, and elevated serum levels of liver enzymes. Complications can include seizures, pneumonitis, rhabdomyolysis, hemorrhage, shock, increased susceptibility to secondary infections, and death (1, 2). HGA occurs predominantly in northeastern and upper Midwestern states, although its geographic range is expanding (9). It is also present in Europe, Scandinavia, and eastern parts of Asia, particularly China, South Korea, and Japan (1). The number of HGA cases reported to the U.S. Centers for Disease Control increased steadily from 348 in 2000, the year the disease became reportable, to 5,672 in 2017, representing a 16.3-fold increase. The incidence of the disease rose 12.8-fold during this time period (http://www.cdc.gov/anaplasmosis/stats/index.html). Seroprevalence studies suggest that HGA is underreported in some areas of endemicity and its true incidence is potentially much higher (10–15). More than 879,000 cases of canine anaplasmosis have been diagnosed in the United States over the past 5 years (http://www.capcvet.org/maps/#2019/all/anaplasmosis/dog/united-states/), which not only signifies its threat as a veterinary disease but also provides sentinel data that underscore the likelihood that the risk for HGA is much greater than indicated by current reporting. HGA can be effectively treated with doxycycline. However, owing to its nonspecific symptoms, it can be difficult to diagnose at presentation, when maximal impact of antibiotic therapy would prevent transition to severe complications. No prophylactic measures for HGA exist (1, 2).

A. phagocytophilum exhibits a biphasic developmental cycle in which its infectious dense-cored (DC) form binds specific surface receptors to promote microbial entry into a host cell-derived vacuole. Within the A. phagocytophilum-occupied vacuole (ApV), the DC form converts to the noninfectious replicative reticulate cell (RC) morphotype that divides by binary fission. Between 28 and 32 h, replication ceases and RCs retransition to DCs that exit to initiate the next wave of infection (16). A. phagocytophilum invasion of mammalian host cells is cooperatively mediated by at least three adhesins, OmpA (outer membrane protein A), AipA (A. phagocytophilum invasion protein A), and Asp14 (14-kDa A. phagocytophilum surface protein) (17–20). Whereas specific lysine and glycine residues of OmpA recognize sialyl-Lewis X and structurally similar glycans (19), functionally essential residues of Asp14 and AipA and the receptors that they target have not been discerned.

Protein disulfide isomerase (PDI) is a member of the thioredoxin superfamily of redox proteins. PDI has thiol-disulfide oxidoreductase, disulfide isomerase, and redox-dependent chaperone activities. It contains four thioredoxin-like domains that consist of two catalytic a and a′ domains separated by noncatalytic b and b′ domains. The two catalytic domains contain the CGHC active site, the cysteines of which are essential for enzymatic activity. PDI is expressed in almost all mammalian tissues and, although it is highly enriched in the endoplasmic reticulum, it is also found in the nucleus, cytoplasm, and at the cell surface (21). Cell surface PDI functions as a reductase (22–24). Neutrophil surface-bound PDI regulates neutrophil αMβ2 integrin-mediated adhesive functions during vascular inflammation (25), and its reductase activity is important for internalization of HIV, Dengue virus, Leishmania chagasi, and Chlamydia trachomatis into host cells (26–33). No other microbial protein that directly interacts with PDI to exploit its reductase activity for invasion has been identified, and the *in vivo* relevance of PDI to infection by any pathogen is unknown.

Herein, we demonstrate that specific C-terminal residues of A. phagocytophilum Asp14 directly interact with host cell surface PDI to bring the enzyme into sufficient proximity such that it reduces bacterial surface disulfide bonds as a prerequisite for optimal entry into host cells. Using myeloid-specific-PDI conditional-knockout (PDI CKO) mice, we confirm that PDI is important for A. phagocytophilum infection *in vivo*. This study dissects a novel microbial cellular invasion mechanism, establishes the first *in vivo* relevance of PDI to infection by any pathogen, and defines the role of the Asp14 binding domain in A. phagocytophilum infection, thereby identifying a new target for preventing or treating granulocytic anaplasmosis.

## RESULTS

### PDI is an Asp14 interacting partner and is important for A. phagocytophilum infection of host cells.

**FIG 1:**
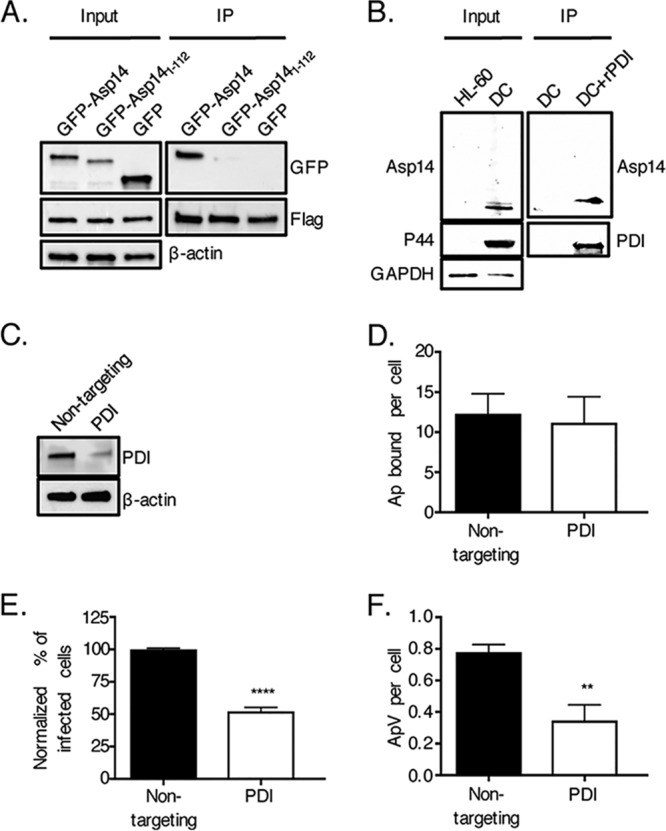
PDI is an Asp14 interacting partner and is important for A. phagocytophilum infection of host cells. (A) Asp14 is capable of binding PDI. HEK-293T cells were transfected to coexpress Flag-PDI and GFP-Asp14, GFP-Asp14_1–112_, or GFP. Input lysates were subjected to Western blotting with GFP and Flag antibodies to verify expression of each protein of interest and with β-actin antibody to confirm that the input lysates contained equivalent amounts of protein. Whole-cell lysates were incubated with Flag antibody-conjugated agarose beads to immunoprecipitate (IP) Flag-PDI and its interacting proteins. The resulting Western blots were probed with Flag antibody to confirm that Flag-PDI was recovered and GFP antibody to determine if Flag-PDI coimmunoprecipitated GFP or either GFP-tagged protein. (B) Native Asp14 interacts with PDI. His-PDI-coupled nickel resin or nickel resin alone was incubated with A. phagocytophilum DC bacteria that had been recovered after sonication of infected HL-60 cells. After the addition of lysis buffer, His-PDI-interacting protein complexes were recovered, followed by Western blot analysis of eluted proteins and input lysates using antibodies against PDI, A. phagocytophilum P44, and human GAPDH. (C to F) PDI is important for A. phagocytophilum infection of but not binding to host cells. HEK-293T cells were treated with nontargeting or PDI-targeting siRNA. (C) PDI knockdown was confirmed by Western blotting. (D to F) Following siRNA treatment, HEK-293T cells were incubated with A. phagocytophilum organisms, followed by assessment of the numbers of bound A. phagocytophilum bacteria (Ap) per cell at 1 h (D) and the percentages of infected cells (E) and numbers of ApVs per cell (F) at 24 h. Data in panels A to C are representative of three independent experiments. Data in panels D to F are representative of three experiments conducted in triplicate. Statistically significant values are indicated. **, *P < *0.01; ****, *P < *0.0001.

A yeast two-hybrid screen using Asp14 as bait and a prey human leukocyte cDNA library identified PDI as a potential Asp14 interacting partner (Table S1 in the supplemental material). Because cell surface PDI contributes to invasion by other intracellular pathogens (26–33), this putative interaction was pursued. The Asp14 residues that are essential for binding to host cell surfaces lie within its C-terminal 12 amino acids, amino acids 113 to 124 (17). Flag-PDI was expressed in HEK-293T cells with green fluorescent protein (GFP)-tagged Asp14 (GFP-Asp14), a GFP-tagged Asp14 sequence comprising residues 1 to 112 (GFP-Asp14_1–112_), or GFP. The cells were lysed and incubated with Flag antibody-coated beads to immunoprecipitate Flag-PDI and interacting proteins. Flag-PDI coimmunoprecipitated GFP-Asp14 but not GFP-Asp14_1–112_ or GFP (Fig. 1A). To confirm if native Asp14 on the A. phagocytophilum surface interacts with PDI, intact DC organisms were incubated with His-PDI coupled to nickel resin. Following the addition of lysis buffer, His-PDI-interacting protein complexes were recovered. Western blot analysis confirmed that native Asp14 binds and precipitates His-PDI (Fig. 1B). Thus, Asp14 interacts with PDI and requires amino acids 113 to 124 to do so.

10.1128/mBio.03141-19.2 TABLE S1 Asp14 interacting partner candidates identified by yeast two-hybrid analysis. Download Table S1, DOCX file, 0.01 MB. Copyright © 2020 Green et al. 2020 Green et al. This content is distributed under the terms of the Creative Commons Attribution 4.0 International license.

To define the relevance of PDI to A. phagocytophilum infectivity, HEK-293T cells were treated with PDI-targeting small interfering RNA (siRNA) or nontargeting siRNA. HEK-293T cells were selected for this purpose because of their amenability to transfection and susceptibility to A. phagocytophilum infection (34–36). PDI knockdown was confirmed via Western blotting (Fig. 1C). PDI knockdown and control cells were incubated with A. phagocytophilum organisms, followed by immunofluorescence microscopy to assess bacterial binding and infection at 1 h and 24 h, respectively. While PDI knockdown had no effect on bacterial binding, it resulted in approximately 2-fold reductions in the percentage of infected cells and number of ApVs per cell (Fig. 1D to F). Therefore, PDI is needed for A. phagocytophilum to optimally infect but not bind to host cells.

### A. phagocytophilum requires PDI to productively infect mice.

**FIG 2:**
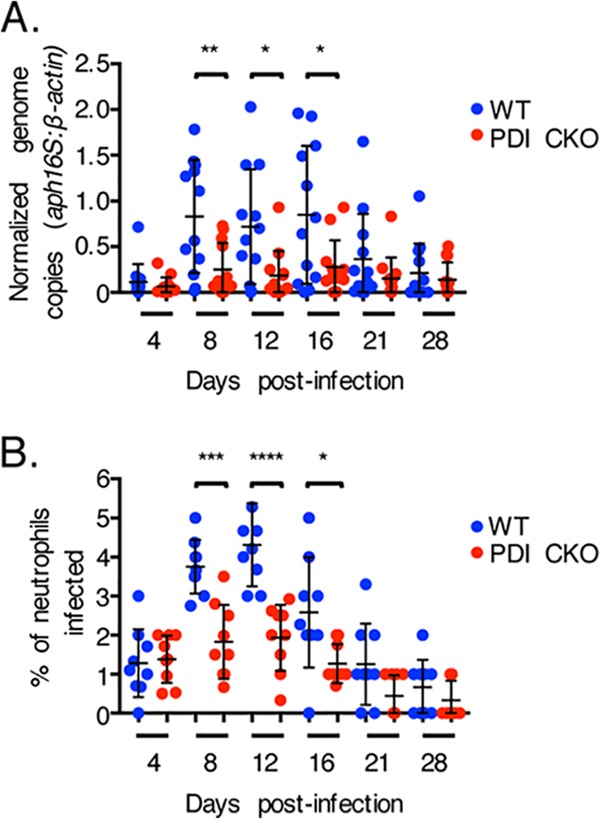
PDI is critical for A. phagocytophilum to productively infect mice. PDI CKO or wild-type (WT) mice were infected with A. phagocytophilum DC organisms. (A) Peripheral blood drawn on the indicated days postinfection was analyzed by qPCR using gene-specific primers. Relative A. phagocytophilum 16S rRNA gene (*aph16S*)-to-murine β-actin DNA levels were determined using the cycle threshold (2^−ΔΔ^*^CT^*) method. Data are the mean normalized bacterial loads ± SD calculated for 12 WT and 12 PDI CKO mice. (B) Peripheral blood samples were also examined for ApV-containing neutrophils by light microscopy. Each dot corresponds to the percentage of A. phagocytophilum-infected neutrophils as determined by examining at least 100 neutrophils per mouse. Data are the mean percentages ± SD determined for nine mice per group. Error bars indicate standard deviations among the samples per time point. Statistically significant values are indicated. *, *P < *0.05; **, *P < *0.01; ***, *P < *0.001; ****, *P < *0.0001.

To determine the relevance of PDI to A. phagocytophilum infection *in vivo*, DC organisms were inoculated into wild-type or myeloid-specific-PDI conditional-knockout (PDI CKO) mice, the latter of which have PDI deleted (≥97% versus the amount in the wild type) in neutrophils and monocytes (25). PDI CKO mice are of the C57BL/6 background, one that is established for studying granulocytic anaplasmosis *in vivo* (37). Peripheral blood samples recovered over a 4-week period were examined by quantitative PCR (qPCR) for the bacterial DNA loads and microscopically examined for neutrophils harboring ApVs. In contrast to that observed for wild-type controls, infection of PDI CKO mice was pronouncedly reduced. On days 8, 12, and 16, which typically present with peak bacteremia levels (38, 39), blood samples from control mice were strongly positive for A. phagocytophilum DNA and had the highest percentages of infected neutrophils, whereas peripheral blood samples from PDI CKO mice carried A. phagocytophilum DNA and ApV-harboring neutrophil loads that were reduced by approximately 2-fold (Fig. 2). As typically observed (38, 39), there was little to no evidence of infection in either mouse strain by day 28. These findings confirm that A. phagocytophilum requires PDI to productively infect neutrophils *in vivo*.

### Cell surface PDI thiol reductase activity is critical for A. phagocytophilum infection.

**FIG 3:**
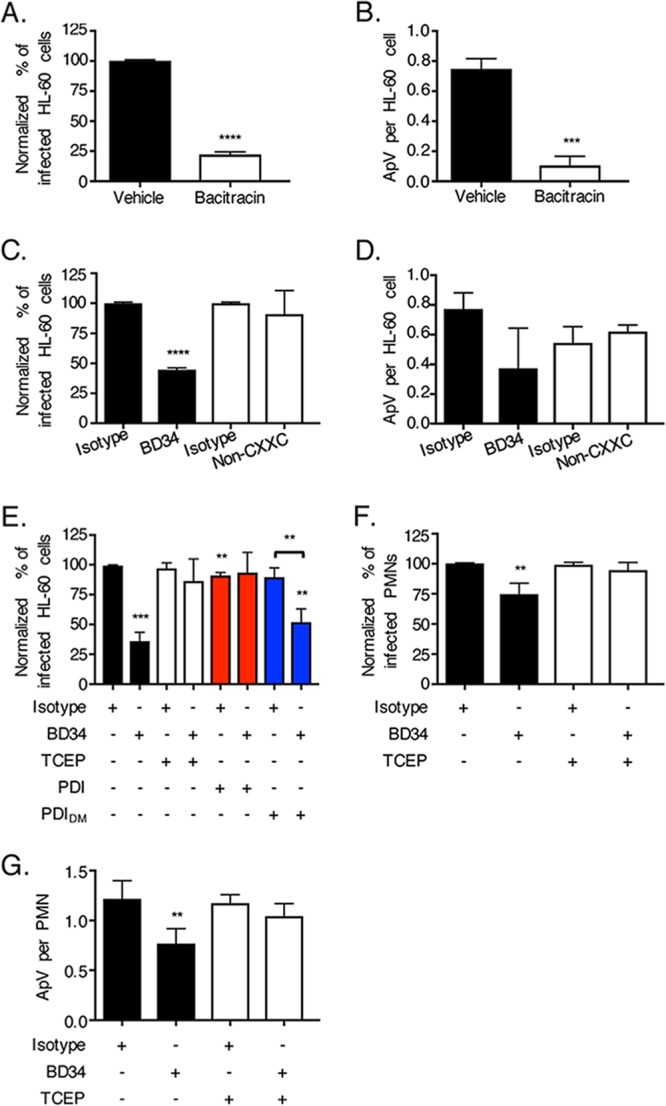
Cell surface PDI reductase activity is important for A. phagocytophilum infection. (A to D) A. phagocytophilum requires PDI enzymatic activity to optimally invade host cells. HL-60 cells were incubated with A. phagocytophilum organisms in the presence of bacitracin or vehicle control (A and B) or antibody BD34, noncatalytically neutralizing PDI antibody (Non-CXXC), or the appropriate isotype control (C and D). At 24 h, the cells were examined by immunofluorescence microscopy for the percentages of infected cells (A and C) and numbers of ApVs per cell (B and D). (E to G) BD34-mediated inhibition of A. phagocytophilum infection is nullified in the presence of recombinant PDI or TCEP. HL-60 cells (E) or neutrophils (polymorphonuclear leukocytes [PMNs]) (F and G) that had been treated with BD34 or isotype control were incubated with A. phagocytophilum in the presence of PDI, PDI_DM_, or TCEP for 30 min, followed by PBS washing. At 24 h, the cells were examined by immunofluorescence microscopy for the percentages of infected cells (E and F) and numbers of ApVs per cell (G). All data are presented as the mean values ± SD from triplicate samples and are representative of experiments performed a minimum of three times. Statistically significant values are indicated. **, *P < *0.01; ***, *P < *0.001; ****, *P < *0.0001. (E) Asterisks above columns indicate statistically significant differences in infection relative to the results for untreated cells, while asterisks over the horizontal bracket denote a statistically significant difference between the levels of infection of BD43- versus isotype control-treated cells following incubation with PDI_DM_.

Because cell surface PDI functions as a disulfide bond reductase (22–24), the question of whether this enzymatic activity is key for A. phagocytophilum infection was examined. First, HL-60 cells were treated with bacitracin. In addition to inhibiting peptidoglycan synthesis, this cyclic polypeptide mixture acts as a membrane-impermeable inhibitor of the reductase activity of PDI and all other cell surface reductases (40, 41). In the presence of bacitracin, A. phagocytophilum infection of HL-60 cells was reduced approximately 4-fold (Fig. 3A and B), whereas bacterial adherence was unaffected (Fig. S1A). In agreement with A. phagocytophilum lacking most peptidoglycan synthesis genes (42), bacitracin treatment of host cell-free bacteria did not alter their infectivity (Fig. S1B). Bacitracin also had no effect on HL-60 cell viability or A. phagocytophilum infection of Ixodes scapularis-derived ISE6 cells (Fig. S1C and D). To determine the exclusive contribution of PDI enzymatic activity to A. phagocytophilum infection, HL-60 cells were treated with monoclonal antibody BD34, which specifically binds to PDI and neutralizes its activity (43), or a noncatalytically neutralizing PDI polyclonal antibody. Neither PDI antibody inhibited A. phagocytophilum cellular adherence (Fig. S1E). Relative to the results for its isotype control, BD34 robustly lessened the percentage of infected cells and bacterial load (Fig. 3C and D). In contrast, nonneutralizing PDI antibody did not.

10.1128/mBio.03141-19.1 FIG S1 Assessment of the effects of bacitracin and antibody BD34 on A. phagocytophilum cellular adherence, A. phagocytophilum and host cell viability, and A. phagocytophilum infection of tick cells. (A) Bacitracin does not inhibit A. phagocytophilum (Ap) binding to host cells. HL-60 cells were incubated with A. phagocytophilum in the presence of bacitracin or vehicle control. At 1 h, the cells were washed to remove unbound bacteria, followed by examination using immunofluorescence microscopy to determine the numbers of bound A. phagocytophilum organisms per cell. (B) Bacitracin treatment of host cell-free A. phagocytophilum bacteria does not alter infectivity. A. phagocytophilum organisms were treated with bacitracin or vehicle, followed by incubation with HL-60 cells. At 24 h, the cells were examined by immunofluorescence microscopy to determine the percentages of infected cells. (C) Bacitracin has no effect on HL-60 cell viability. HL-60 cells treated with bacitracin for 1 h were assessed for survival using trypan blue exclusion. (D) Bacitracin has no effect on A. phagocytophilum infection of ISE6 cells. ISE6 cells were incubated with A. phagocytophilum in the presence of bacitracin or vehicle control. At 24 h, the cells were examined by immunofluorescence microscopy for the percentages of infected cells. (E and F) Antibody BD34 does not inhibit A. phagocytophilum binding to HL-60 cells or neutrophils. HL-60 cells (E) or neutrophils (polymorphonuclear leukocytes [PMNs]) (F) were treated with antibody BD34, noncatalytically neutralizing PDI antibody (Non-CXXC), or the appropriate isotype control, followed by incubation with A. phagocytophilum organisms. At 1 h, the cells were washed to remove unbound bacteria, followed by immunofluorescence microscopy to enumerate the numbers of bound A. phagocytophilum organisms per cell. All data are presented as the mean values ± SD from triplicate samples and are representative of experiments performed a minimum of three times. Download FIG S1, PDF file, 0.1 MB. Copyright © 2020 Green et al. 2020 Green et al. This content is distributed under the terms of the Creative Commons Attribution 4.0 International license.

The membrane-impermeable reducing agent tris(2-carboxyethyl)phosphine–HCl (TCEP) or recombinant PDI will complement enzymatically defective cell surface PDI (26, 44). To further confirm that PDI-mediated disulfide bond reduction is important for A. phagocytophilum infection, HL-60 cells that had been treated with BD34 to neutralize cell surface PDI enzymatic activity or with the isotype control and subsequently incubated with DC bacteria were exposed to TCEP, vehicle control, recombinant wild-type PDI, or recombinant enzymatically defective double mutant (DM) PDI (PDI_DM_) (PDI with changes of C to S at positions 36, 39, 383, and 386 [PDI_C36S,C39S,C383S,C386S_]) (44). Here, the driving rationale was that TCEP or recombinant wild-type PDI would reduce disulfide bonds on the surfaces of both bacterial and host cells to complement BD34-mediated inactivation of host cell surface PDI. The cells were examined for ApVs at 24 h postinfection. TCEP and wild-type PDI but not PDI_DM_ restored the ability of A. phagocytophilum to efficiently infect BD34-treated cells (Fig. 3E). Similarly, BD34 had no inhibitory effect on A. phagocytophilum binding to human neutrophils (Fig. S1F), but it reduced infection in a TCEP-rescuable manner (Fig. 3F and G). These data demonstrate that PDI reduction of disulfide bonds present on the bacterial and/or host cell surface is essential for optimal A. phagocytophilum infection.

### PDI-mediated thiol reduction of the A. phagocytophilum surface, but not the host cell surface, promotes infection.

**FIG 4:**
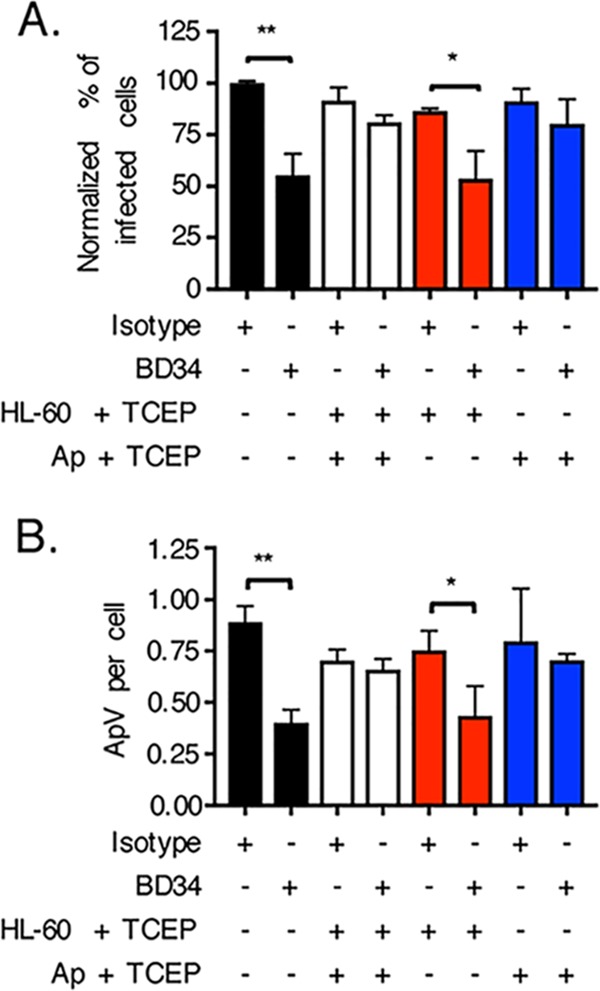
PDI-mediated disulfide bond reduction of the A. phagocytophilum surface but not the host cell surface is key for infection. HL-60 cells were incubated with antibody BD34 to catalytically inhibit cell surface PDI or with isotype control antibody. The HL-60 cells were then treated with TCEP for 30 min to chemically reduce host cell surface disulfide bonds, followed by PBS washing and subsequent incubation with A. phagocytophilum organisms. Alternatively, A. phagocytophilum DC organisms were treated with TCEP for 30 min to reduce bacterial cell surface disulfide bonds, followed by PBS washing and subsequent incubation with BD34- or isotype control-treated HL-60 cells. At 24 h, the HL-60 cells that had been exposed to A. phagocytophilum (Ap) under each condition were examined by immunofluorescence microscopy for the percentages of infected cells (A) and numbers of ApVs per cell (B). Data are presented as the mean values ± SD from triplicate samples and are representative of individual experiments performed three times. Statistically significant values are indicated. *, *P < *0.05; **, *P < *0.01.

To differentiate whether the key surface protein(s) that PDI reduces is host or bacterial, the TCEP rescue experiment was repeated with two additional conditions. First, BD34-treated HL-60 cells were incubated with TCEP, followed by phosphate-buffered saline (PBS) washing and then the addition of A. phagocytophilum DC bacteria, so that only host cell surface disulfide bonds were reduced. Second, A. phagocytophilum DC organisms were exposed to TCEP, washed with PBS, and incubated with BD34 treated HL-60 cells such that only bacterial surface disulfide bonds were reduced. TCEP treatment of A. phagocytophilum but not HL-60 cells rescued the bacterium’s ability to infect BD34-treated host cells (Fig. 4). Therefore, PDI-catalyzed disulfide bond reduction of one or more A. phagocytophilum surface proteins is critical for bacterial infectivity.

### Host cell surface thioredoxin-1 activity can benefit A. phagocytophilum infection of host cells.

**FIG 5:**
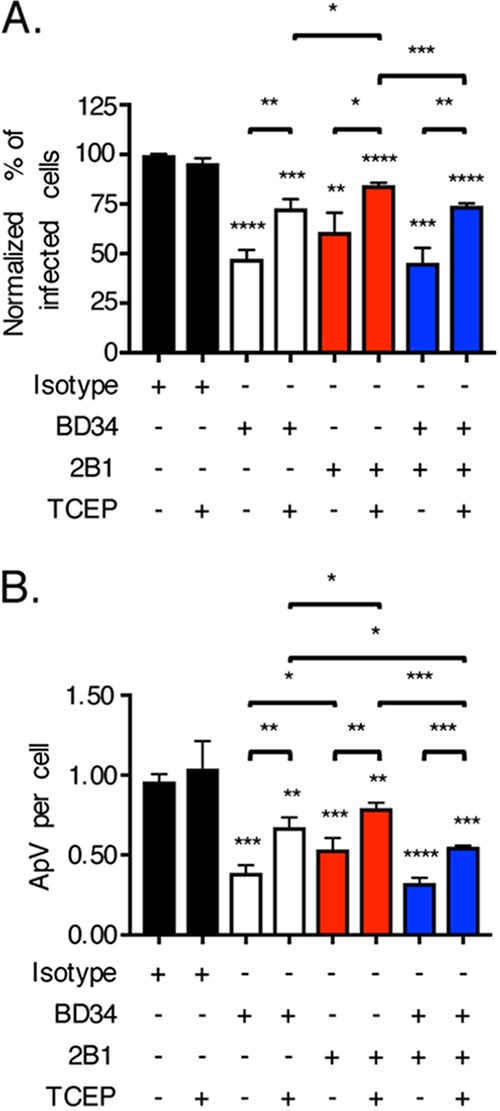
Host cell surface thioredoxin-1 reductase activity can contribute to A. phagocytophilum infection. HL-60 cells were incubated with A. phagocytophilum organisms in the presence of PDI-specific antibody BD34, thioredoxin-specific antibody 2B1, both BD34 and 2B1, or isotype control antibody followed by treatment with TCEP or vehicle. At 24 h, the cells were examined by immunofluorescence microscopy for the percentages of infected cells (A) and numbers of ApVs per cell (B). Data are presented as the mean values ± SD from triplicate samples and are representative of experiments performed three separate times. Statistically significant values are indicated. *, *P < *0.05; **, *P < *0.01; ***, *P < *0.001; ****, *P < *0.0001. Asterisks above columns indicate statistically significant differences in infection relative to the results for isotype control-treated cells, while asterisks above horizontal brackets denote statistically significant differences between the indicated column pairs.

In addition to PDI, other reductases are present at mammalian cell surfaces, including thioredoxin-1 (Trx1), an enzyme whose reductase activity, like that of PDI, contributes to HIV cellular entry (28, 29, 45–49). 2B1 is a Trx1-specific monoclonal antibody that inhibits Trx1 activity-dependent HIV cellular entry (29). To determine if cell surface Trx1 reductase activity contributes to A. phagocytophilum infection and to compare its relative contribution to that of PDI, DC organisms were incubated in the presence or absence of TCEP with HL-60 cells that had been treated with 2B1, BD34, or both antibodies. 2B1 and BD34 reduced the percentages of cells infected comparably (Fig. 5). The difference in reduction in ApVs per cell achieved by BD34 versus 2B1 was statistically significant, albeit modestly (Fig. 5B). Inhibition achieved by both antibodies together was similar to that achieved by either independently (Fig. 5). TCEP partially reversed A. phagocytophilum infectivity irrespective of cell surface reductase antibody treatment. Thus, cell surface PDI and Trx1 benefit A. phagocytophilum infection of host cells comparably.

### The Asp14 C-terminal binding domain is important for A. phagocytophilum to co-opt host cell surface PDI reductase activity.

**FIG 6:**
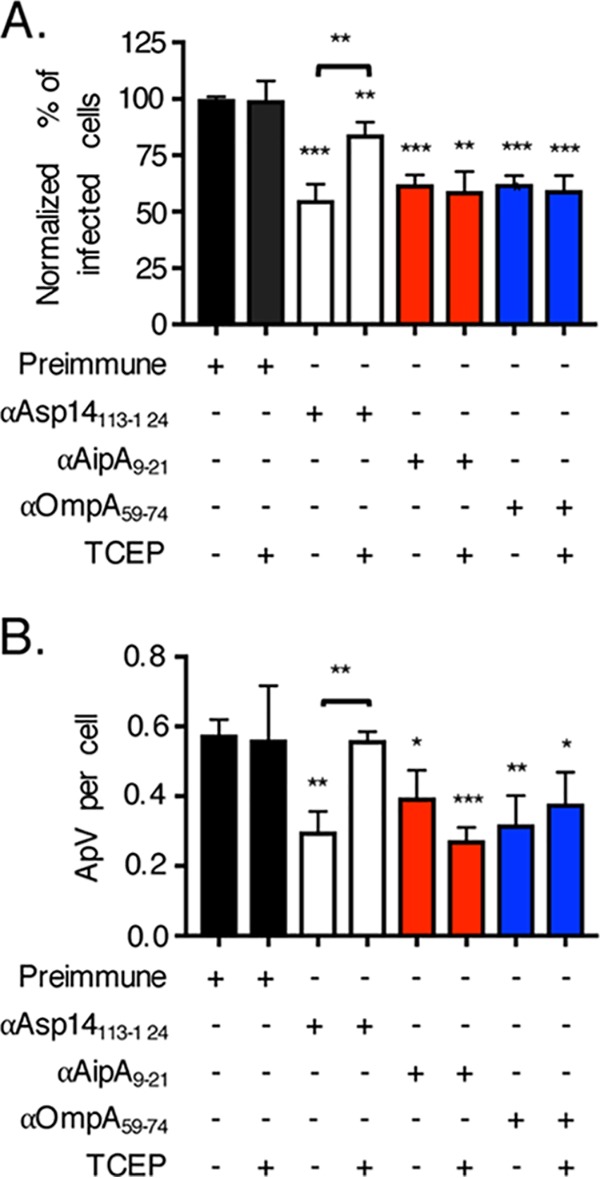
The Asp14 C terminus is important for the ability of A. phagocytophilum to exploit host cell surface disulfide bond reductase activity. A. phagocytophilum bacteria were treated with rabbit preimmune serum or rabbit antiserum (α) specific for Asp14_113–124_, AipA_9–21_, or OmpA_59–74_, followed by incubation with HL-60 cells in the continued presence of antiserum with or without TCEP. At 24 h, the cells were examined by immunofluorescence microscopy for the percentages of infected cells (A) and numbers of ApVs per cell (B). Data are presented as the mean values ± SD from triplicate samples and are representative of experiments performed three separate times. Statistically significant values are indicated. *, *P < *0.05; **, *P < *0.01; ***, *P < *0.001; ****, *P < *0.0001. Asterisks above columns indicate statistically significant differences in levels of infection relative to the results for preimmune serum-treated cells, while asterisks over the horizontal brackets denote a statistically significant difference between the levels of infection of Asp14_113–124_ antiserum-treated cells following incubation in the absence or presence of TCEP.

Asp14 functions cooperatively with OmpA and AipA to mediate A. phagocytophilum infection of host cells (19). The binding domains of all three adhesins have been identified (17–20). While Asp14 C-terminal binding domain residues 113 to 124 are essential for interacting with PDI (17), whether OmpA or AipA participates in PDI exploitation is unclear. Therefore, the question of whether the ability of A. phagocytophilum to co-opt PDI reductase activity for infection is exclusively linked to the Asp14 binding domain or also involves those of OmpA and AipA was examined. DC bacteria were treated with preimmune serum or antiserum specific for Asp14_113–124_, AipA_9–21_, or OmpA_59–74_, followed by incubation with HL-60 cells in the presence or absence of TCEP. As reported previously (17–20), each receptor binding domain-targeting antiserum significantly reduced A. phagocytophilum infection in the absence of TCEP (Fig. 6). Notably, TCEP nullified the inhibitory effect of anti-Asp14_113–124_ antiserum, but not that of either anti-AipA_9–21_ or anti-OmpA_59–74_ antiserum. Thus, of these three adhesins, only Asp14 is involved in interacting with and exploiting the enzymatic activity of PDI, and it does so by virtue of its C-terminal binding domain.

### Identification of Asp14 C-terminal residues that are critical for binding PDI.

**FIG 7:**
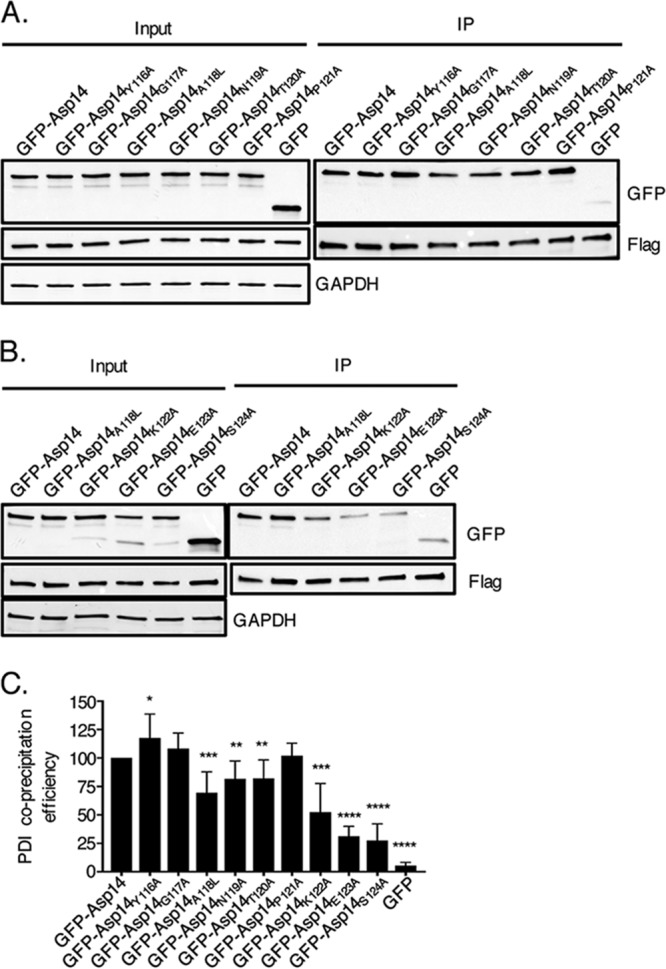
Identification of the Asp14 C-terminal residues that are essential for interacting with PDI. (A and B) HeLa cells were transfected to coexpress Flag-PDI and GFP, GFP-Asp14, or GFP-Asp14 bearing the indicated amino acid substitution. Input lysates were subjected to Western blotting with GFP and Flag antibodies to verify expression of each protein of interest and with GAPDH (glyceraldehyde-3-phosphate dehydrogenase) antibody to confirm that the input lysates contained equivalent amounts of protein. Whole-cell lysates were incubated with Flag antibody-conjugated agarose beads to immunoprecipitate Flag-PDI and its interacting proteins. The resulting Western blots were probed with Flag antibody to confirm that Flag-PDI was recovered and GFP antibody to determine if Flag-PDI coimmunoprecipitated GFP or GFP-tagged Asp14 protein. (C) The efficiency with which Flag-PDI coprecipitated GFP or GFP-Asp14 fusion proteins was determined as follows. The densitometric signal of coprecipitated GFP or GFP-Asp14 fusion protein was divided by that of immunoprecipitated Flag-PDI. The resulting ratio was divided by the quotient of the densitometric signal of input GFP fusion protein divided by that of input Flag-PDI. The resulting efficiency value was normalized by dividing it by the coprecipitation value determined for GFP-Asp14 and multiplied by 100%. The mean normalized coprecipitation efficiency ± SD from three to seven replicates per condition was determined. Statistically significant values relative to the results for GFP-Asp14 are indicated. *, *P < *0.05; **, *P < *0.01; ***, *P < *0.001; ****, *P < *0.0001.

To delineate Asp14 C-terminal residues that are key for PDI binding, the Flag-PDI coimmunoprecipitation assay was performed using HEK-293T cells expressing Flag-PDI and GFP or GFP-tagged Asp14 or Asp14 proteins bearing amino acid substitutions in the C-terminal PDI binding domain. Asp14 residues of interest were replaced with alanine, except for alanine 118, which was replaced with leucine. The efficiencies with which Flag-PDI coimmunoprecipitated GFP-Asp14_Y116A_, GFP-Asp14_G117A_, and GFP-Asp14_P121A_ were comparable to the efficiency of its coimmunoprecipitation of GFP-Asp14, suggesting that Asp14 residues Y116, G117, and P121 are dispensable for binding PDI (Fig. 7). However, Flag-PDI pulldown of GFP-tagged Asp14_A118L_, Asp14_N119A_, and Asp14_T120A_ was reduced by 18% to 30%, indicating that A118, N119, and T120 each contribute to the interaction. The interaction was decreased by 48% for Asp14_K122A_, 69% for Asp14_E123A_, and 73% for Asp14_S124A_, indicating that Asp14 K122, E123, and S124 contribute most significantly to PDI binding.

**FIG 8:**
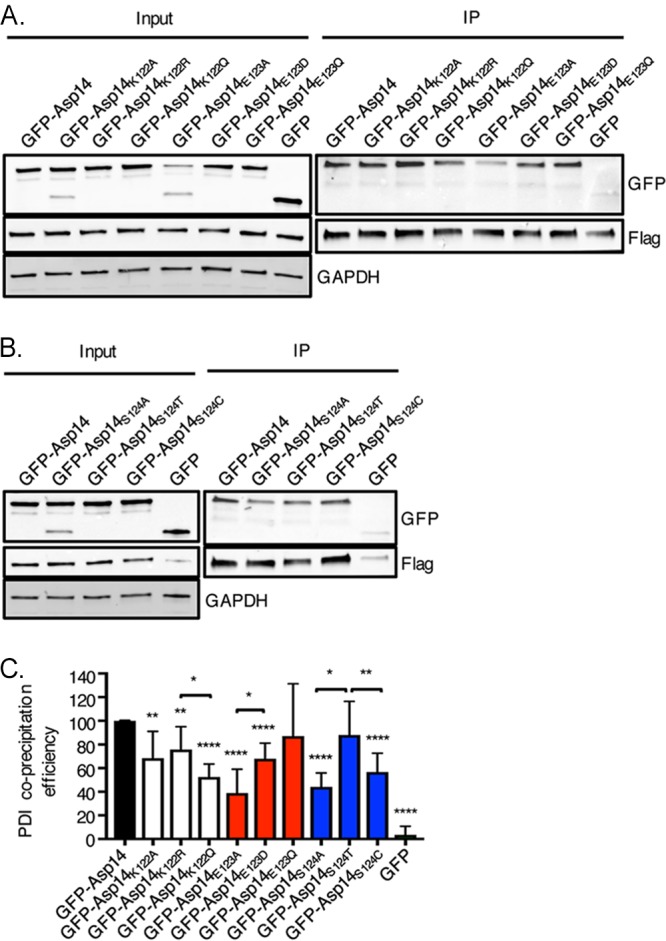
The relative contributions of K122, E123, and S124 R group properties to Asp14 PDI binding efficiency. (A and B) HeLa cells were transfected to coexpress Flag-PDI and GFP, GFP-Asp14, or GFP-Asp14 bearing the indicated amino acid substitution. Input lysates were subjected to Western blotting with GFP and Flag antibodies to confirm protein expression and with GAPDH antibody to confirm that the input lysates contained equivalent amounts of protein. Whole-cell lysates were incubated with Flag antibody-conjugated agarose beads to immunoprecipitate Flag-PDI and its interacting proteins. The resulting Western blots were probed with Flag antibody to confirm that Flag-PDI was recovered and GFP antibody to determine if Flag-PDI coimmunoprecipitated GFP or GFP-tagged Asp14 protein. (C) The efficiency with which Flag-PDI coprecipitated GFP or GFP-Asp14 fusion proteins was determined as described above. The mean normalized coprecipitation efficiency ± SD from three to six replicates per condition was determined. Statistically significant values are indicated. *, *P < *0.05; **, *P < *0.01; ****, *P < *0.0001. Asterisks above columns indicate statistically significant differences relative to the results for GFP-Asp14, while asterisks over horizontal brackets denote statistically significant differences between the indicated column values.

To determine the differential contributions of K122, E123, and S124 charge, size, and/or polarity to PDI binding, the interaction efficiencies of Flag-PDI with GFP-tagged Asp14 and Asp14 proteins having various substitutions for these residues were compared. Replacement of K122 with the smaller and hydrophobic alanine or with glutamine, which is of comparable size to lysine and polar but uncharged, reduced the Asp14-PDI interaction similarly (Fig. 8). Substitution with the positively charged but larger arginine resulted in a more modest reduction in pulldown efficiency than did the glutamine substitution. Swapping E123 for alanine, which reduces size and eliminates both polarity and negative charge at this position, was considerably more disruptive than substitution with the polar, negatively charged, and slightly smaller aspartate. Changing E123 to glutamine, which is also polar and similarly sized but neutral, was ineffectual. Replacing S124 with alanine, which eliminates polarity and slightly reduces size, or with cysteine, which is similar in size and hydrophobic but less so than alanine, reduced the interaction similarly. Synonymous serine-to-threonine substitution at this position had no effect. Based on these results, the following can be concluded. First, the size of E123 and the polarity of it and S124 are vital for the Asp14-PDI interaction. Second, while its contribution is comparatively less than that of E123 or S124, the K122 positive charge likely mediates an ionic interaction that aids Asp14 binding to PDI.

## DISCUSSION

Entry into host cells is essential for the survival of obligate intracellular microbes and their ability to cause disease. Dissecting this fundamental step can reveal novel protective and therapeutic targets. This study demonstrates that Asp14 binds mammalian cell surface PDI to bring A. phagocytophilum in sufficient proximity to the enzyme that it reduces bacterial surface disulfide bridges, which in turn facilitates optimal pathogen invasion. Inhibiting this mechanism by knocking down PDI cellular levels, neutralizing PDI catalytic activity, and treating with Asp14-blocking antibody each reduces infection by 50%. Deletion of PDI in myeloid cells considerably reduces the pathogen load in mice, thereby further establishing the importance of PDI to A. phagocytophilum infectivity and providing the first confirmation of its relevance to *in vivo* infection by any pathogen. Moreover, Asp14 is the first microbial protein identified that directly engages PDI to orchestrate pathogen entry. Multiple *Chlamydia* species bind cell surface PDI, and Chlamydia trachomatis has been reported to co-opt PDI reductive activity for invasion, but no responsible chlamydial protein has been identified (26, 50, 51). Asp14-mediated entry is thematically distinct from that of the well-characterized HIV entry mechanism in that the virus does not directly bind a cell surface reductase. Rather, HIV gp120 binds host CD4 to bring gp120 close to PDI, Trx1, and/or glutaredoxin-1 such that its disulfide bonds are reduced to induce the conformational changes necessary for mediating membrane fusion (27–29, 31, 45, 47–49, 52).

Asp14_113–124_ was previously demonstrated to be critical for A. phagocytophilum infection, but the underlying reason was unknown (17). This report fills the knowledge gap by demonstrating that the domain contains residues that are essential for binding PDI. Asp14 amino acids A118, N119, and T120 contribute to the interaction, K122 more so, and E123 and S124 are critical. Ehrlichia chaffeensis, Ehrlichia canis, and Ehrlichia ruminantium, which are obligate intracellular pathogens in the family *Anaplasmataceae* with A. phagocytophilum, carry Asp14 orthologs. Although the roles of these ehrlichial proteins are undefined, they conspicuously contain a lysine-aspartate pair within their C-terminal-most residues (17). The studies herein set the stage for investigating these orthologs’ relevance to *Ehrlichia* species cellular invasion.

Bacitracin reduces A. phagocytophilum infection of myeloid cells more pronouncedly than BD34 antibody or PDI knockdown. The Asp14-PDI interaction could promote disulfide bond reduction of bacterial outer membrane proteins by bringing them in sufficient proximity to other host cell surface reductases in addition to PDI. In support of this possibility, antibody-mediated neutralization of cell surface PDI and Trx1 each inhibits A. phagocytophilum infection comparably in a TCEP-rescuable manner, and both enzymes, as well as cell surface glutaredoxin-1, can reduce HIV gp120 to promote viral entry (27–29, 31, 45, 47–49, 52). However, simultaneously inactivating PDI and Trx1 does not exert an additive inhibitory effect on A. phagocytophilum and bacitracin does not eliminate infection, which hints that Asp14-mediated entry is complemented by an invasion route that is independent of disulfide bond reduction. Indeed, A. phagocytophilum engagement of sialyl-Lewis X-capped P-selectin glycoprotein ligand-1, presumably by OmpA, leads to Syk-mediated phosphorylation of ROCK1, which contributes to bacterial entry (18, 19, 53, 54). Thus, Asp14 and OmpA likely perform distinct and independent but cooperative roles in bacterial invasion. Bacitracin had no effect on A. phagocytophilum infection of ISE6 cells, indicating that it co-opts cell surface disulfide bond reduction to invade mammalian but not tick cells. Consistent with this observation, A. phagocytophilum does not transcribe *asp14* in unfed Ixodes scapularis ticks but upregulates its expression during the tick bloodmeal that transmits the bacterium into mammals (17).

PDI is dispensable for bacterial adherence, which, when considered with prior reports (17–20), suggests that adhesin-receptor interactions, including those between OmpA and sialyl-Lewis X and AipA and its unknown binding partner, facilitate bacterial docking to the host cell surface to enable Asp14-mediated invasion. Of the known A. phagocytophilum adhesins/invasins, however, Asp14 is the only one that exploits host cell surface reductive activity, as TCEP restored the infectivity of bacteria treated with antiserum specific for Asp14_113–124_ but not that of bacteria treated with antiserum specific for either OmpA_59–74_ or AipA_9–21_.

Antibody-mediated catalytic inactivation of neutrophil surface PDI inhibits A. phagocytophilum infection in a TCEP-rescuable manner, but not as robustly as for HL-60 cells. This discrepancy is likely due to an inherent limitation of the *in vitro* model. A. phagocytophilum inhibits apoptosis (55–57). Due to the inhibitory effect of BD34 on the bacterium’s ability to invade cells, we rationalize that most BD34-treated neutrophils did not get infected and consequently apoptosed, leaving behind a surviving population consisting mainly of infected cells. The importance of PDI to A. phagocytophilum invasion of neutrophils can be inferred from the recalcitrance of PDI CKO neutrophils to infection *in vivo* despite the fact that the number of circulating neutrophils is unaltered in these mice (25). Notably, however, PDI CKO neutrophils do not efficiently arrest on endothelium (25). Clustering of A. phagocytophilum-infected neutrophils in tissues, which is dependent on the leukocytes’ ability to tether to endothelial cell surfaces, leads to an increased bacterial burden in mice (58). Thus, the inability of PDI CKO neutrophils to arrest on endothelium could possibly contribute to the observed reduction of A. phagocytophilum infection *in vivo*.

The A. phagocytophilum surface protein(s) that are disulfide bond reduced following the Asp14-PDI interaction are unknown. Because Asp14 and mature OmpA each have only one cysteine (17, 18), neither would possess intramolecular disulfide bridges. The predicted extracellular regions of AipA have a total of five cysteines, including C24, which is adjacent to the adhesin binding domain encompassed by residues 9 to 21 (20). To fully decipher the Asp14-orchestrated invasion mechanism, it will be important to determine the A. phagocytophilum outer membrane protein that becomes disulfide bond reduced and its role in infection. Because cell-associated reductases drive conformational changes in the surface proteins of intracellular pathogens that lead to microbial invasion, many reductase inhibitors have been evaluated for their anti-infective properties, primarily against HIV. These include 5,5′-dithiobis-2-nitrobenzoic acid, trivalent arsenic compounds, juniferdin, and auranofin, all of which potently block HIV entry but are impractical for therapeutic use due to their high cytotoxicity (59). However, due to the importance of PDI to A. phagocytophilum infection *in vivo* and since antibody against the Asp14_113–124_ PDI binding domain inhibits invasion of host cells as effectively as catalytically neutralizing PDI itself, vaccination to elicit a humoral immune response against this domain would be expected to noncytotoxically protect against granulocytic anaplasmosis by blocking the Asp14-PDI interaction and preventing the thiol-disulfide exchange that is key for infection.

Due to the potential severity of granulocytic anaplasmosis, understanding A. phagocytophilum cellular invasion is highly important. By elucidating how Asp14 mediates infection, this study identifies a new target for protecting against A. phagocytophilum and establishes a thematic approach that could be applied to prevent diseases caused by intracellular pathogens that bind PDI or other cell surface reductases for entry.

## MATERIALS AND METHODS

### Cell lines and cultivation of A. phagocytophilum.

Uninfected and A. phagocytophilum strain NCH-1-infected human promyelocytic HL-60 cells were cultured as described previously (38). Human embryonic kidney HEK-293T and Ixodes scapularis embryonically derived ISE6 cells were cultured as described previously (38, 60).

### Antibodies, chemicals, reagents, plasmid constructs, and recombinant proteins.

Antisera against Asp14_113–124_, OmpA_59–74_, and AipA_9–21_ were generated previously (19, 20). Commercial antibodies were BD34 (BD Biosciences), 2B1 (ThermoFisher Scientific, Rockford, IL), anti-PDI antibody (MilliporeSigma, St. Louis, MO), anti-β-actin antibody (Santa Cruz Biotechnology, Dallas, TX), anti-GAPDH (glyceraldehyde-3-phosphate dehydrogenase) antibody (Santa Cruz), anti-GFP antibody (Invitrogen), anti-Flag antibody (Invitrogen), Alexa Fluor 488-conjugated goat anti-mouse IgG and goat anti-rabbit IgG (Invitrogen), and horseradish peroxidase-conjugated goat anti-mouse IgG and anti-rabbit IgG (Cell Signaling Technology, Danvers, MA). Bacitracin and M2 Flag affinity resin were procured from Alfa Aesar (Ward Hill, MA) and MilliporeSigma, respectively. TCEP, Lipofectamine 2000, and protein A/G agarose resin were obtained from Thermo Fisher Scientific and Dharmafect from Dharmacon (Lafayette, CO). All plasmids are listed in Table S2 in the supplemental material. Vector pBMH carrying a mammalian-codon-optimized DNA sequence encoding full-length Asp14 (residues 1 to 124, locus APH_0248; UniProtKB identification number [ID] Q2GL86_ANAPZ), which was originally determined for A. phagocytophilum strain HZ (61) and is identical to that of strain NCH-1, was provided by Biomatik (Wilmington, DE). pUC57 vectors carrying mammalian-codon-optimized DNA sequences encoding Asp14_G117A_, Asp14_A118L_, Asp14_N119A_, Asp14_T120A_, or Asp14_P121A_ were provided by Genscript (Piscataway, NJ). DNA sequences encoding all additional Asp14 proteins bearing amino acid substitutions were PCR amplified using mammalian-codon-optimized Asp14 plasmid as the template and primers listed in Tables S2 and S3. Each sequence was PCR amplified using primers bearing EcoRI or SalI sites (Tables S2 and S3), followed by restriction enzyme digestion and ligation into pEGFP-C1 (Clontech, Palo Alto, CA) as described previously (62). The gene sequence for human PDI (*P4HB*; UniProtKB ID P07237) was PCR amplified from plasmid ccsbBroadEn_01138 (DNASU Plasmid Repository, Tempe, AZ) (63) using primers in Table S2 and restriction ligated into pCMV 3xFLAG 7.1 (MilliporeSigma) as described previously (62) to generate pFlag-PDI. Constructs encoding His-tagged PDI and PDI_DM_, described elsewhere (44), were transformed into Escherichia coli BL21(DE3) for protein production and purification by immobilized metal affinity chromatography as described previously (64, 65).

10.1128/mBio.03141-19.3 TABLE S2 Plasmids used in this study. Download Table S2, DOCX file, 0.02 MB. Copyright © 2020 Green et al. 2020 Green et al. This content is distributed under the terms of the Creative Commons Attribution 4.0 International license.

### Yeast two-hybrid analysis.

ULTImate yeast two-hybrid analysis was performed by Hybrigenics Services (Paris, France). The mammalian-codon-optimized DNA sequence encoding A. phagocytophilum Asp14 was cloned into pB27 as an N-terminal fusion with LexA (N-LexA-Asp14-C). The construct was introduced into yeast as bait and screened by mating with yeast harboring a randomly primed human leukocyte cDNA library (prey). Prey fragments from positively selected clones were PCR amplified, sequenced, and identified using the NCBI GenBank database and Basic Local Alignment Search Tool (http://blast.ncbi.nlm.nih.gov/blast). The predicted biological score was calculated to assess the reliability of each interaction, ranging from the highest (A score) to the lowest (E score) probability of specificity between two proteins (66).

### Immunoprecipitation.

HEK-293T cells grown in six-well plates to 80% confluence were cotransfected with 2 μg each of plasmid encoding GFP or GFP-tagged Asp14, Asp14_113–124_, or Asp14 bearing amino acid substitutions (Table S2) together with 2 μg plasmid encoding Flag-PDI as described previously (67). At 16 h, the cells were lysed and Flag-PDI and interacting proteins were precipitated using Flag-affinity agarose resin (MilliporeSigma) as described previously (68). Eluates were resolved by SDS-PAGE in 4-to-20% mini-Protean gels (Bio-Rad, Hercules, CA) as described previously (62). Western blot analyses were performed as described previously (67) using GFP and Flag tag primary antibodies at a 1:1,000 dilution and horseradish peroxidase (HRP)-conjugated secondary antibodies at a 1:10,000 dilution. Input lysates were subjected to Western blot analysis using β-actin antibody at a 1:2,500 dilution to confirm that immunoprecipitations were performed using equivalent amounts of lysate per sample. The efficiency with which Flag-PDI coprecipitated GFP or GFP-Asp14 fusion proteins was calculated as follows. First, the densitometric signal of coprecipitated GFP or GFP-Asp14 fusion protein was divided by that of immunoprecipitated Flag-PDI. Second, the resulting ratio was divided by the quotient of the densitometric signal of input GFP protein divided by that of input Flag-PDI. Third, the resulting efficiency value was normalized by dividing by the coprecipitation value determined for GFP-Asp14 and multiplied by 100%. The mean normalized coprecipitation efficiency ± standard deviation (SD) of at least three experiments per condition and statistical significance were determined.

### His-PDI precipitation of native Asp14.

A. phagocytophilum DC bacteria were recovered from 2 × 10^7^ heavily infected (≥90%) HL-60 cells by sonication as described previously (69). DC organisms were incubated with a 50-μl suspension of His-Bind resin (MilliporeSigma) in the presence or absence of 4 μg of His-PDI in a final volume of 1.1 ml PBS at 4°C for 3 h with constant rotation. The resin was pelleted by centrifugation at 1,000 × *g* for 1 min at 4°C, followed by the addition of 100 μl of lysis buffer and incubation on ice for 30 min. The resin was washed three times with 1 ml lysis buffer each time with centrifugation at 1,000 × *g* for 1 min at 4°C followed by resuspension in Laemmli buffer with freshly added β-mercaptoethanol. Eluates and input lysates were examined by Western blot analysis.

### A. phagocytophilum cellular infection assays.

Human neutrophils were isolated as described previously (38). Investigations using human neutrophils were conducted according to the principles expressed in the Helsinki Declaration, and informed consent was obtained from all subjects. The protocol (HM11407) for obtaining donor blood has been reviewed and approved by the Virginia Commonwealth University Institutional Review Board with respect to scientific content and compliance with applicable research and human subject regulations. To determine the relevance of cell surface reductase activity to A. phagocytophilum binding to and infection of host cells, HL-60 cells, ISE6 cells, or neutrophils were incubated with 3 mM bacitracin in Iscove's modified Dulbecco's medium (Invitrogen) containing 10% (vol/vol) fetal bovine serum (Gemini Bioproducts, West Sacramento, CA) alone or with 10 μg ml^−1^ BD34, anti-PDI, or isotype control in IMDM-10 for 1 h at 37°C, followed by incubation with DC organisms (18, 60). Triplicate samples were analyzed by indirect immunofluorescence microscopy at 60 min to determine the number of bound A. phagocytophilum organisms per cell or at 24 h to determine the number of A. phagocytophilum-occupied vacuoles (ApVs) per cell and the percentage of infected cells (18). The percentage of infected cells for each condition was normalized to that for vehicle- or isotype control-treated cells. In some cases, bacitracin- or antibody-treated HL-60 cells were incubated with A. phagocytophilum in the presence of 40 μg ml^−1^ His-PDI or His-PDI_DM_ for 30 min at 37°C prior to processing for immunofluorescence microscopy. In certain instances, antibody-treated HL-60 cells or neutrophils were incubated with DC bacteria followed by the addition of TCEP to a final concentration of 0.01 mM for 30 min at 37°C prior to PBS washing and processing for immunofluorescence microscopy. In other cases, either antibody-treated HL-60 cells or untreated A. phagocytophilum bacteria were incubated in IMDM-10 containing 0.01 mM TCEP at 37°C for 30 min followed by PBS washing and subsequent incubation with each other. To determine which A. phagocytophilum adhesin co-opts cell surface PDI, DC bacteria were treated with 100 μg ml^−1^ of heat-killed antiserum specific for Asp14_113–124_, OmpA_59–74_, or AipA_9–21_ or preimmune serum for 1 h, followed by incubation with HL-60 cells in the presence or absence of 0.01 mM TCEP at 37°C for 30 min prior to assessment for infection. To determine the effect of knocking down PDI on infection, HEK-293T cells were treated with 5 μM On-Target plus human *P4HB* or nontargeting control siRNA (Dharmacon) and target knockdown was confirmed (34) prior to incubation with A. phagocytophilum.

### Mouse studies.

The generation and breeding of myeloid-specific-PDI conditional-knockout (PDI CKO) CKO mice have been described previously (25). Six- to 12-week-old PDI CKO or age- and sex-matched C57BL/6 control mice (Jackson Laboratories, Bar Harbor, ME) were intraperitoneally inoculated with 1 × 10^8^ A. phagocytophilum DC organisms (39). Quantification of the peripheral blood A. phagocytophilum burden was determined as described previously (39). All animal research was performed under the approval of the Institutional Animal Care and Use Committee at Virginia Commonwealth University (protocol number AM10220).

### Statistical analyses.

Statistical analyses were performed using Prism 7.0 (GraphPad, San Diego, CA). Student’s *t* test was used to test for significant differences among pairs. Statistical significance was set at *P* values of <0.05.

10.1128/mBio.03141-19.4 TABLE S3 Oligonucleotides used in this study. Download Table S3, DOCX file, 0.01 MB. Copyright © 2020 Green et al. 2020 Green et al. This content is distributed under the terms of the Creative Commons Attribution 4.0 International license.
