# Supplementary material for: Binding of Host Cell Surface Protein Disulfide Isomerase by Anaplasma phagocytophilum Asp14 Enables Pathogen Infection
Source: mBio. 2020 Jan 28;11(1):e03141-19. doi: 10.1128/mBio.03141-19 (PMC6989111; doi:10.1128/mBio.03141-19)
Supplement: TABLE S1 [file mBio.03141-19-st001.docx]

**TABLE S1.** Asp14 candidate interacting partners identified via yeast two-hybrid analysis

| Candidate | Name and function | Known subcellular localization | PBS*^a^* |
| --- | --- | --- | --- |
| AHCYL1 | Adenosylhomocysteine hydrolase-like protein 1; regulates cellular processes including epithelial fluid secretion, mRNA processing, and DNA replication. | Plasma membrane, endoplasmic reticulum, cytosol | C |
| IKBKG | NFκB essential modulator; a component of the IKK core complex that positively regulates NFκB activity. | Cytosol, nucleus | C |
| P4HB | Protein disulfide isomerase; oxidizes, reduces, and isomerizes disulfide bonds to promote native protein conformations and regulate protein function. | Plasma membrane,  endoplasmic reticulum | B |
| PPP1R15A | Protein phosphatase 1 regulatory subunit 15A; recruits protein phosphatase PP1 to dephosphorylate target proteins. | Endoplasmic reticulum, mitochondria | C |
| XRCC6 | X-ray repair cross-complementing protein 6; a single stranded DNA helicase that plays a role in DNA double-strand break repair and recombination. | Nucleus | C |
| ZMYM5 | Zinc finger, MYM-type protein 5; transcriptional regulator | Nucleus | B |

*^a^*The predicted biological score (PBS) was calculated for each candidate protein to assess the reliability of each predicted interaction, scores range from the highest probability of specificity (score of A) to the lowest probability of specificity (score of E) between two proteins.
