# Supplementary figures and images for: Binding of Host Cell Surface Protein Disulfide Isomerase by Anaplasma phagocytophilum Asp14 Enables Pathogen Infection
Source: mBio. 2020 Jan 28;11(1):e03141-19. doi: 10.1128/mBio.03141-19 (PMC6989111; doi:10.1128/mBio.03141-19)

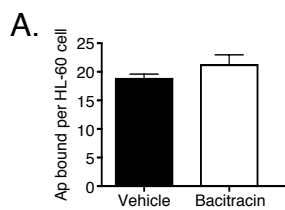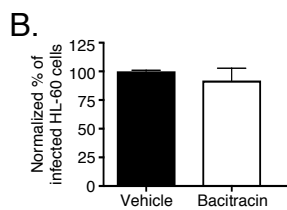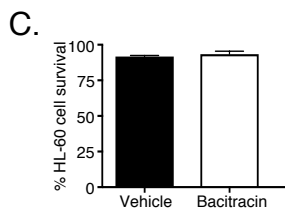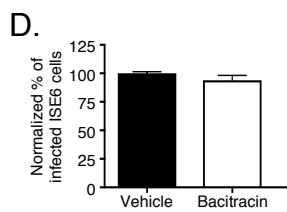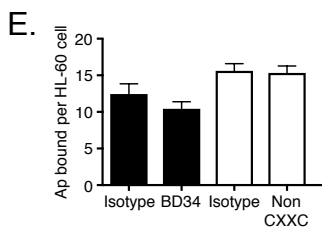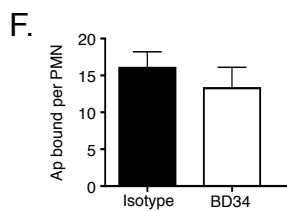

Supplement: FIG S1 [file mBio.03141-19-sf001.pdf]
