# Supplementary material for: Binding of Host Cell Surface Protein Disulfide Isomerase by Anaplasma phagocytophilum Asp14 Enables Pathogen Infection
Source: mBio. 2020 Jan 28;11(1):e03141-19. doi: 10.1128/mBio.03141-19 (PMC6989111; doi:10.1128/mBio.03141-19)
Supplement: TABLE S2 [file mBio.03141-19-st002.docx]

**Table S2.** Plasmids used in this study

| Construct name | Parent plasmid | Insert Sequence | Source, reference, or method generated | Primers utilized |
| --- | --- | --- | --- | --- |
| ccsbBroadEn_01138 | pENTR223 | Human *P4HB* cDNA | DNASU plasmid repository |  |
| pFlag-PDI | pCMV 3xFLAG 7.1 | Human *P4HB* cDNA | PCR amplification, restriction-ligation | *P4HB*-4EcoRI F  *P4HB*-1524SalI R |
| pHis-PDI | pET-15B | Rat *P4HB* cDNA | Reference 44 |  |
| pHis-PDI_DM_ | pET-15B | Rat *P4HB* cDNA | Reference 44 |  |
| pBMH-Asp14 | pBMH | Mammalian codon-optimized *asp14* | Synthesized |  |
| pGFP-Asp14 | pEGFP-C1 | Mammalian codon-optimized *asp14* | PCR amplification, restriction-ligation | *asp14*-4EcoRI F  *asp14*-372SalI R |
| pGFP-Asp14 _1-112_ | pEGFP-C1 | Mammalian codon optimized *asp14_1-112_* | PCR amplification, restriction-ligation | *asp14*-4EcoRI F  *asp14*-336SalI R |
| pAsp14_G117A_ | pUC57 | Mammalian codon optimized *asp14_G117A_* | Synthesized |  |
| pGFP-Asp14_G117A_ | pEGFP-C1 | Mammalian codon optimized *asp14_G117A_* | Restriction-ligation |  |
| pAsp14_A118L_ | pUC57 | Mammalian codon optimized *asp14_A118L_* | Synthesized |  |
| pGFP-Asp14_A118L_ | pEGFP-C1 | Mammalian codon optimized *asp14_A118L_* | Restriction-ligation |  |
| pAsp14_N119A_ | pUC57 | Mammalian codon optimized *asp14_N119A_* | Synthesized |  |
| pGFP-Asp14_N119A_ | pEGFP-C1 | Mammalian codon optimized *asp14_N119A_* | Restriction-ligation |  |
| pAsp14_T120A_ | pUC57 | Mammalian codon optimized *asp14_T120A_* | Synthesized |  |
| pGFP-Asp14_T120A_ | pEGFP-C1 | Mammalian codon optimized *asp14_T120A_* | Restriction-ligation |  |
| pAsp14_P121A_ | pUC57 | Mammalian codon optimized *asp14_P121A_* | Synthesized |  |
| pGFP-Asp14_P121A_ | pEGFP-C1 | Mammalian codon optimized *asp14_P121A_* | Restriction-ligation |  |
| pGFP-Asp14_Y116A_ | pEGFP-C1 | Mammalian codon optimized *asp14_Y116A_* | PCR amplification, restriction-ligation | *asp14*-4EcoRI F  *asp14*-Y116ASalI R |
| pGFP-Asp14_K122A_ | pEGFP-C1 | Mammalian codon optimized *asp14_K122A_* | PCR amplification, restriction-ligation | *asp14*-4EcoRI F  *asp14*-K122ASalI R |
| pGFP-Asp14_K122Q_ | pEGFP-C1 | Mammalian codon optimized *asp14_K122Q_* | PCR amplification, restriction-ligation | *asp14*-4EcoRI F  *asp14*-K122QSalI R |
| pGFP-Asp14_K122R_ | pEGFP-C1 | Mammalian codon optimized *asp14_K122R_* | PCR amplification, restriction-ligation | *asp14*-4EcoRI F  *asp14*-K122RSalI R |
| pGFP-Asp14_E123A_ | pEGFP-C1 | Mammalian codon optimized *asp14_E123A_* | PCR amplification, restriction-ligation | *asp14*-4EcoRI F  *asp14*-E123ASalI R |
| pGFP-Asp14_E123D_ | pEGFP-C1 | Mammalian codon optimized *asp14_E123D_* | PCR amplification, restriction-ligation | *asp14*-4EcoRI F  *asp14*-E123DSalI R |
| pGFP-Asp14_E123Q_ | pEGFP-C1 | Mammalian codon optimized *asp14_E123Q_* | PCR amplification, restriction-ligation | *asp14*-4EcoRI F  *asp14*-E123QSalI R |
| pGFP-Asp14_S124A_ | pEGFP-C1 | Mammalian codon optimized *asp14_S124A_* | PCR amplification, restriction-ligation | *asp14*-4EcoRI F  *asp14*-S124ASalI R |
| pGFP-Asp14_S124C_ | pEGFP-C1 | Mammalian codon optimized *asp14_S124C_* | PCR amplification, restriction-ligation | *asp14*-4EcoRI F  *asp14*-S124CSalI R |
| pGFP-Asp14_S124T_ | pEGFP-C1 | Mammalian codon optimized *asp14_S124T_* | PCR amplification, restriction-ligation | *asp14*-4EcoRI F  *asp14*-S124TSalI R |
