# Supplementary material for: Binding of Host Cell Surface Protein Disulfide Isomerase by Anaplasma phagocytophilum Asp14 Enables Pathogen Infection
Source: mBio. 2020 Jan 28;11(1):e03141-19. doi: 10.1128/mBio.03141-19 (PMC6989111; doi:10.1128/mBio.03141-19)
Supplement: TABLE S3 [file mBio.03141-19-st003.docx]

**Table S3.** Oligonucleotides used in this study

| Designation*^a^* | Sequence (5’-3’) |
| --- | --- |
| *Ap16S* F | TGTAGGCGGTTCGGTAAGTTAAAG |
| *Ap16S* R | GCACTCATCGTTTACAGCGTG |
| *asp14-*4EcoRI F | ATCGGAATTCATACCATTAGCTCCTTGGAAGAGCATTTCGGTGGTGTA |
| *asp14*-336SalI R | CGATGTCGACTTATTCAAGCTTGAGAATCCTTCTTTTA |
| *asp14-*372SalI R | CGATGTCGACTTAGCTTTCTTTAGGAGTATTGGCACCGTAAACAGCCCT |
| *asp14-*Y116ASalI R | CGATGTCGACTTAGCTTTCTTTAGGAGTATTGGCACCCGCAACAGCCCT |
| *asp14-*K122ASalI R | CGATGTCGACTTAGCTTTCCGCAGGAGTATTGGCACCGTAAACAGCCCT |
| *asp14-*K122QSalI R | CGATGTCGACTTAGCTTTCCTGAGGAGTATTGGCACCGTAAACAGCCCT |
| *asp14-*K122RSalI R | CGATGTCGACTTAGCTTTCCCTAGGAGTATTGGCACCGTAAACAGCCCT |
| *asp14-*E123ASalI R | CGATGTCGACTTAGCTCGCTTTAGGAGTATTGGCACCGTAAACAGCCCT |
| *asp14-*E123DSalI R | CGATGTCGACTTAGCTGTCTTTAGGAGTATTGGCACCGTAAACAGCCCT |
| *asp14-*E123QSalI R | CGATGTCGACTTAGCTCTGTTTAGGAGTATTGGCACCGTAAACAGCCCT |
| *asp14-*S124ASalI R | CGATGTCGACTTACGCTTCTTTAGGAGTATTGGCACCGTAAACAGCCCT |
| *asp14-*S124CSalI R | CGATGTCGACTTACCTTTCTTTAGGAGTATTGGCACCGTAAACAGCCCT |
| *asp14-*S124TSalI R | CGATGTCGACTTAAGTTTCTTTAGGAGTATTGGCACCGTAAACAGCCCT |
| *β-actin* F | AGAGGGAAATCGTGCGTGAC |
| *β-actin* R | CAATAGTGATGACCTGGCCGT |
| *Lys-Cre* F | CCCAGAAATGCCAGATTACG |
| *Lys-Cre* R | CTTGGGCTGCCAGAATTTCTC |
| *pdi-*4*EcoRI* F | ATCGGAATTCACTGCGCCGCGCTCTGCTG |
| *pdi-*1524*SalI* R | ATCGGTCGACCAGTTCATC TTTCACAGCTTTCTG |
| *P4HB* F | GGCTGAGTTATCTGGTGATTGACCAATG |
| *P4HB* R | TCCAGGCTCCACAAAAATTCCTTTAGC |

*^a^*F and R refer to primers that bind to the sense or antisense strand, respectively.
